# Supplementary material for: Deep Learning for Fully Automatic Tumor Segmentation on Serially Acquired Dynamic Contrast-Enhanced MRI Images of Triple-Negative Breast Cancer
Source: Cancers (Basel). 2023 Oct 2;15(19):4829. doi: 10.3390/cancers15194829 (PMC10571741; doi:10.3390/cancers15194829)
Supplement: Supplementary file 1 [file cancers-15-04829-s001.zip › cancers-2576206-supplementary.pdf]

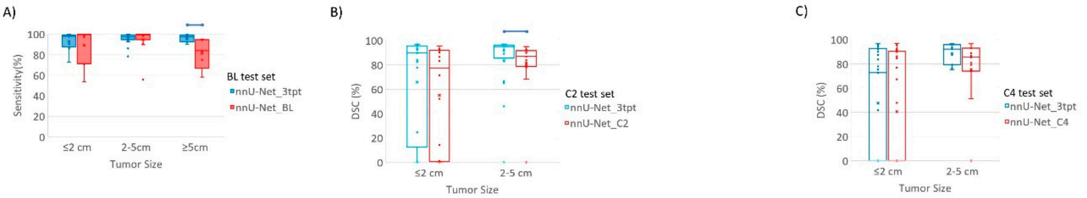

D)

Model	Tumor Size	Sensitivity		
		Median	Quartile	
			Q1	Q3
nnUnet_3tpt	≤2 cm	98	88	99
	2-5cm	98	95	99
	≥5cm	98	93	99
nnUnet_BL	≤2 cm	99	72	100
	2-5cm	99	99	100
	≥5cm	84	71	95

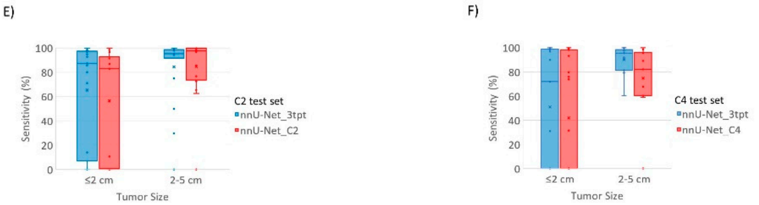

G)

Model	Tumor Size	DSC			Sensitivity		
		Median	Quartile		Median	Quartile	
			Q1	Q3		Q1	Q3
nnUnet_3tpt	≤2 cm	90	19	95	87	11	97
	2-5cm	95	86	96	95	92	99
nnUnet_C2	≤2 cm	77	1	92	83	1	91
	2-5cm	87	79	92	97	75	100

H)

Model	Tumor Size	DSC			Sensitivity		
		Median	Quartile		Median	Quartile	
			Q1	Q3		Q1	Q3
nnUnet_3tpt	≤2 cm	73	0	92	72	0	99
	2-5cm	92	81	96	96	83	98
nnUnet_C4	≤2 cm	0	0	90	0	0	98
	2-5cm	85	75	92	82	62	96

Supplementary Figure S1. Segmentation performance of nnU-Net models by tumor size across different datasets. (**A**) Tumor segmentation performance of nnU-Net models on baseline (BL), (**B,E**) after two cycles (C2), (**C,F**) and after four cycles (C4) test sets across different tumor sizes. Horizontal bar indicates significant difference in paired Wilcoxon signed rank test ($p<0.05$). (**D**), the detailed quantitative results used for the boxplots in (**A**). (**G**), the detailed quantitative results used for the boxplots in (**B**) and (**E**). (**H**), the detailed quantitative results used for the boxplots in (**C**) and (**F**).

- nnU-Net model training configuration and procedure

The source code of nnU-Net was downloaded from https://github.com/MIC-DKFZ/nnUNet. All trainings used the default set-up[36], and important parameters included the following: data augmentation was implemented using the batchgenerators framework that was included in nnU-Net (a series of augmentations involving spatial, color, noise, and crop processes); the training length was fixed at 1000 epochs, with 250 training iterations per epoch; and the learning rate was 0.01 initially and then was gradually reduced using the "polyLR" schedule[55]. All training used the 2D nnU-Net framework and full-resolution 3D nnU-Net framework and then ensembled these frameworks for inferences. A five-fold cross-validation was performed on the development dataset (training + validation, randomly split at 4:1).
